# Impact of High Power Interference Sources in Planning and Deployment of Wireless Sensor Networks and Devices in the 2.4 GHz Frequency Band in Heterogeneous Environments

**DOI:** 10.3390/s121115689

**Published:** 2012-11-12

**Authors:** Peio López Iturri, Juan Antonio Nazábal, Leire Azpilicueta, Pablo Rodriguez, Miguel Beruete, Carlos Fernández-Valdivielso, Francisco Falcone

**Affiliations:** Electrical and Electronic Engineering Department, Universidad Pública de Navarra, 31006 Pamplona, Spain; E-Mails: peio.lopez@unavarra.es (P.L.I.); juanantonio.nazabal@unavarra.es (J.A.N.); leyre.azpilicueta@unavarra.es (L.A.); pablo.rodriguez@unavarra.es (P.R.); miguel.beruete@unavarra.es (M.B.); carlos.fernandez@unavarra.es (C.F.-V.)

**Keywords:** electromagnetic interference, microwave oven, ray launching, electromagnetic field simulation, ZigBee, 802.15.4, wireless sensor networks, 2.4 GHz ISM band, smart home

## Abstract

In this work, the impact of radiofrequency radiation leakage from microwave ovens and its effect on 802.15.4 ZigBee-compliant wireless sensor networks operating in the 2.4 GHz Industrial Scientific Medical (ISM) band is analyzed. By means of a novel radioplanning approach, based on electromagnetic field simulation of a microwave oven and determination of equivalent radiation sources applied to an in-house developed 3D ray launching algorithm, estimation of the microwave oven's power leakage is obtained for the complete volume of an indoor scenario. The magnitude and the variable nature of the interference is analyzed and the impact in the radio link quality in operating wireless sensors is estimated and compared with radio channel measurements as well as packet measurements. The measurement results reveal the importance of selecting an adequate 802.15.4 channel, as well as the Wireless Sensor Network deployment strategy within this type of environment, in order to optimize energy consumption and increase the overall network performance. The proposed method enables one to estimate potential interference effects in devices operating within the 2.4 GHz band in the complete scenario, prior to wireless sensor network deployment, which can aid in achieving the most optimal network topology.

## 1. Introduction

Nowadays microwave ovens are very popular and can be found in almost every home and building. The heating source of these residential microwave ovens is based on a magnetron tube. The generated electromagnetic field does not remain in the oven's cavity, due to the fact that there are losses due mainly to power leakage from around the microwave oven's front door [1–4]. This leakage can be considered in practical terms as an interference source in the 2.4 GHz Industrial Scientific Medical (ISM) band [5,6], depending strongly on the reflection coefficient and filtering properties of the metallic mesh embedded in the microwave oven door, which exhibit a frequency-dependent response.

In parallel, there is a wide variety of communication systems and protocols, such as digital radio communication systems, Bluetooth, 802.11 and ZigBee that operate in the 2.4 GHz ISM band which can be potentially interfered by the power leakage of microwave ovens [7–10]. Due to the existence of this leakage power, it is important analyze the performance of these devices operating in the 2.4 GHz ISM in the presence of microwave ovens, since this interference can completely block services provided by such wireless systems. This effect is becoming more relevant due to fact that the number of existing applications based mainly on ZigBee wireless sensor networks operating in the 2.4 GHz band in indoor scenarios is rapidly growing (e.g., home automation and monitoring, energy management, health monitoring and lighting) [11–17].

**Figure 1.**
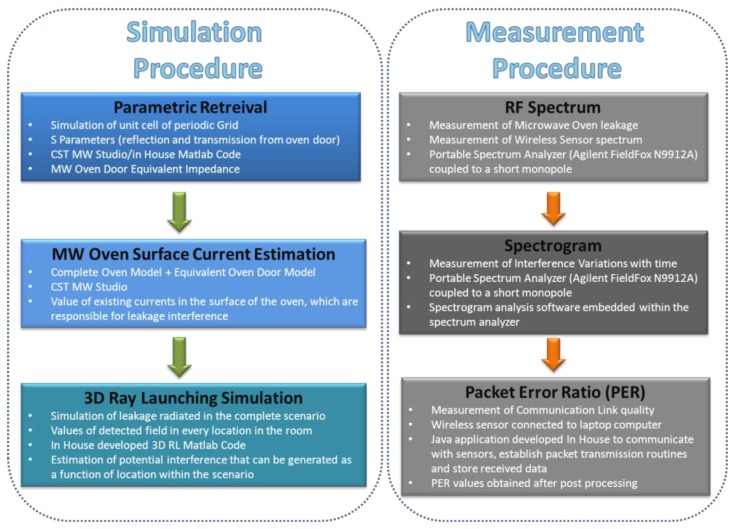
Description of the different simulation and measurement procedures applied.

The negative impact that microwave oven power leakage has on the quality of a ZigBee communication in buildings has been demonstrated in the literature [18–21]. It is worth noting that even though it is a known interference source, characterization of such interference and adequate radioplanning analysis within an environment with wireless systems under operation has not been reported. This is mainly due to the fact that such modeling requires computationally intensive electromagnetic calculations. In this work the coexistence between a ZigBee wireless sensor network, implemented with Digi XBee Pro devices, operating at 2.4 GHz and a real domestic microwave oven has been analyzed, following a novel simulation approach in order to characterize the complete indoor scenario. For this purpose, full wave electromagnetic results, obtained with the aid of CST Microwave Studio have been used to simulate the behavior of the microwave oven power leakage. From these previous results, in order to analyze the propagation of the fadiofrequency (RF) leakage power in an indoor scenario, an equivalent model of the oven has been created *ad-hoc* to be applied in combination with in-house ray launching simulation software developed in our research group, in order to fully estimate interference in the indoor scenario. Finally, a real scenario has been set up to perform measurements of leakage radiated power and its impact on transmission quality of ZigBee channels of operating wireless sensor nodes. The simulation as well as measurement procedure described in this paper and detailed in the following sections is schematically depicted in Figure 1.

The paper is divided in the following sections: Section 2 describes the simulation procedure for the localized interference source (*i.e.*, microwave oven leakage). Section 3 describes the simulation procedure for the complete indoor scenario, based on 3D ray launching algorithm, coupled with the equivalent radiation source derived in the previous section. Section 4 describes the measurement results and analysis of operating radiolinks between wireless sensors in terms of interference, followed by the last section of concluding remarks.

## 2. Characterization of the Microwave Oven as an Interference Source

There are a variety of techniques which can be used for simulate the electromagnetic behavior of a microwave oven, based on finite element approach, finite-difference time-domain method (FDTD) or other 3D electromagnetic approaches [22–25]. These techniques allow one to characterize the behavior of the microwave oven, in terms of efficiency in heating process as well as to analyze power leakage, within close vicinity of the microwave oven device. However, none of these previous approaches is employed in order to simulate how this leakage power is propagated in an indoor scenario, thus becoming an effective interference source. In this paper, CST Microwave Studio, based on Finite Integration Time Domain full wave simulation, has been used for modeling the microwave oven, as an initial step in order to identify the equivalent interference source within the complete scenario. Due to the fact that full wave simulation techniques are computationally demanding, the goal is to obtain information on the equivalent radiation sources in order to combine these results with efficient deterministic 3D ray launching algorithm developed in-house. By following this approach, the overall result is to reduce the computational complexity of the simulation scenario, speeding the simulation process and enabling the analysis of the full simulation scenario.

**Figure 2.**
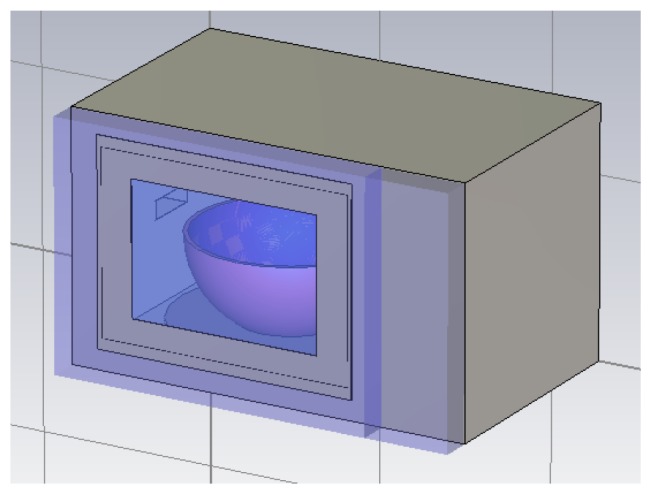
Schematic view of the microwave oven simulation model. The rectangular waveguide and the heated object (a porcelain bowl in the center of the cavity) are shown.

The microwave oven that has been used in this work is the BMG20-4 model of the Bluesky brand. The simulation model has been developed using the real dimensions of the oven. The cavity dimensions are X = 285 mm, Y = 270 mm and Z = 205 mm. The resulting simulation model is shown in Figure 2, in which a porcelain bowl filled with water (which will be used as the object to heat) above the turntable has also been included.

The microwave oven heating source is a magnetron, which has been modeled by a rectangular waveguide, fed by a rectangular waveguide port. In Figure 2 the waveguide coupled in the microwave oven cavity wall can be seen. The dimensions of the waveguide have been calculated to propagate the fundamental TE10 mode at 2.45 GHz, which are length a = 612 mm and height b = 20 mm. The oven has three power levels of operation, with a maximum level of 800 Watt. In the simulations that have been performed, the power of the port that feeds the waveguide has been set at 1Watt in order to have the possibility of normalizing it to the microwave oven power level. This consideration has been taken into account later on, when these results are employed in the ray launching simulation of the complete indoor scenario, presented in the following section.

The electromagnetic properties of the constitutive materials of the microwave oven have a great influence on the results [24], due mainly to their electric permittivity, as well as the effect of losses, given by the loss tangent at the given frequency of operation. In this work non-magnetic materials have been used, which is the normal case in real microwave oven enclosing elements. Therefore, the boundary conditions, electromagnetic properties of the materials and the dimensions of the constitutive elements of the model have been selected accurately and according to the real materials that have been used in the experimental measurements. The material of the cavity walls, the waveguide and the external box are defined as Perfect Electric Conductors (PEC), a valid approximation given the frequency of operation under consideration. The bowl is porcelain, filled with normal water and the turntable is made of lossy glass. The external part of the microwave oven front door is Plexiglas. The parameters of the materials employed are presented in Table 1.

**Figure 3.**
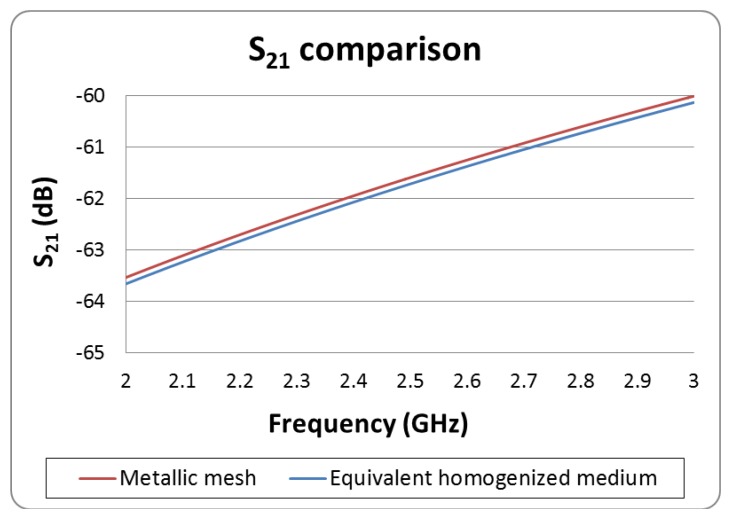
Comparison and validation between the S21 obtained by 3D EM simulation of the oven front door metallic mesh and the S21 parameter of the new material.

The main goal at this point is to correctly determine the distributions of currents in the vicinity of the microwave oven, in order to estimate the overall power leakage from the microwave oven within the indoor scenario later on. The perforated metal walls inside the cavity can be replaced by solid PEC sheets in the model [26]. In order to characterize the power leakage mechanism, the front door and its metallic mesh are the key elements, due to the fact that non-metallic elements and physical gaps can be present. In this work, the structure of the metallic mesh embedded in the microwave oven front door has been simulated independently, as a Frequency Selective Surface unit cell. This approach has been followed in order to give a valid description of the effect of the oven front door (which is one of the main contributors to RF leakage), while allowing reduction in computational complexity and hence simulation time. Once the metallic mesh has been simulated, Scattering parameters (*i.e.*, transmission as well as reflection characteristics of impinging waves, characterized by their voltage values) are obtained and used to perform an equivalent media retrieval from both parameters (S11, which corresponds to the reflection coefficient and S21, which corresponds to the transmission coefficient and in this particular case, determines the power leakage from the front door) to create an effective homogeneous slab which exhibits frequency-dependent properties [27]. This slab has been characterized as a material with the effective permittivity and permeability data obtained in the retrieval procedure. Later on, further simulations have been performed with the slab as a unit cell to obtain the S-parameters of the new effective material, which will be embedded in the oven front door. These new S-parameters have been compared with the previous ones to verify that the new material used in the simulation is valid. In Figure 3 the comparison between the transmission S21 parameters is depicted, indicating that the process has been successful and the obtained data is therefore valid.

**Figure 4.**
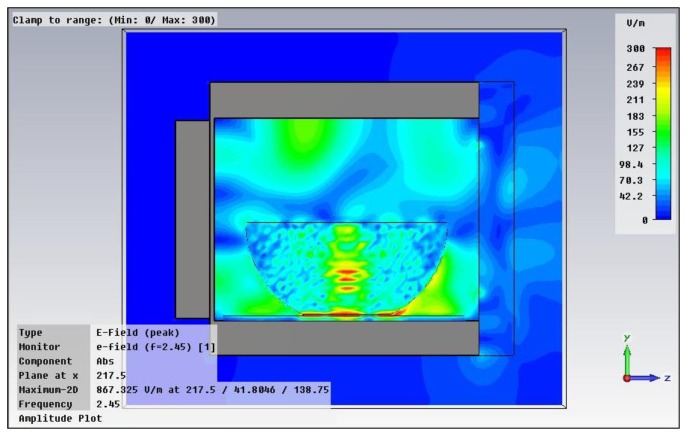
A lateral cut taken from the simulation of the microwave oven. The amplitude of the electric field is represented, in which the oven front door is located on the right hand side of the figure.

Once the characterization of the embedded mesh in the microwave oven front door has been performed and the new effective material has been introduced, the complete oven structure has been simulated by means of 3D full wave simulation. Due to the complexity of the microwave oven and the heated bowl, the number of mesh-cells increases to 12,188,160, leading to a computationally intensive calculation. Figure 4 shows a lateral cut taken from the simulated system, in which the power leakage at the oven door can be seen.

The electromagnetic field distribution calculated outside the oven is necessary for the later 3D ray launching simulations, which will allow the estimation of interference effect within the complete simulation scenario and will be described in the following section.

To summarize, the simulation procedure in this section is the following:

- Full wave electromagnetic simulation: in order to compute scattering parameters, the equivalent value of the surface impedance of the microwave door (which is the main leakage source of the microwave oven) has been obtained. The door is modeled as a periodic surface composed by unit elements that constitute the metallic grid with holes. Once these scattering parameters have been computed, an in-house parameter fitting algorithm implemented in Matlab has been employed to obtain this surface impedance value.
- Full wave electromagnetic simulation: in order to identify the radiation sources within the surface of the microwave oven. For this purpose, a detailed model of a microwave oven and a container with water has been modeled using CST Microwave Studio Time Domain Solver. In this simulation, the microwave door has been modeled with surface impedance value previously calculated. This is done in order to increase the simulation speed, due to the fact that the grid of the microwave door has very small dimensions as compared with the complete microwave oven, leading to a very fine simulation mesh. With our approximation (*i.e.*, including the microwave oven equivalent surface impedance model), simulation time is drastically reduced without compromising accuracy. In order to verify the validity of this approximation, a full wave simulation result of the complete grid has been computed and compared with the model, with very similar results.

## 3. Ray Launching Simulation

The previous simulation results show the values of power leakage in the close vicinity of the microwave oven. However, for radioplanning purposes it is necessary to estimate interference levels within an indoor scenario, such as a household or office location, in which one or more Wireless Sensor Networks can be deployed. Application of 3D full wave electromagnetic techniques is not feasible, due to the large computational effort they require, given the size of the proposed scenario. It is necessary therefore to apply alternative simulation methods to finally obtain interference estimations within the complete indoor scenario.

Traditionally, empirical methods (such as COST-231, Walfish-Bertoni, *etc.*) have been used for initial coverage estimation. They give rapid results, but require calibration based on measurements in order to give an adequate fit of the results, based on initial regression methods, as well as limited consideration of precise material parameters or multipath propagation. On the other hand, deterministic methods are based on numerical approaches to the resolution of Maxwell's equations, such as ray launching and ray tracing or full wave simulation techniques. These methods are precise, but are time consuming to inherent computational complexity. As a midpoint, methods based on geometrical optics, for radio planning calculations with strong diffractive elements, offer a reasonable trade-off between precision and required calculation time. The approach followed in this work is to perform electromagnetic propagation analysis around the microwave oven with the aid of an in-house implemented 3D ray launching algorithm. Ray launching is a technique based on Geometrical Optics (GO) and Geometrical Theory of Diffraction (GTD) that can easily be applied as an approximate method for estimating the levels of high-frequency electromagnetic fields. The underlying principle in GO is that energy can be considered to be radiated through infinitesimally small tubes, often called rays. These rays lie along the direction of propagation and travel in straight lines. Therefore, signal propagation can be modeled via ray propagation. By using the concept of ray tracing, rays can be launched from a transmitter location and the interaction of the rays can be described using the well-known theory of refraction and reflection and interactions with the neighboring environment. The rays considered in GO are only direct, reflected and refracted rays, giving rise to abrupt transition areas in the boundaries of the regions where these rays exist. To complement the GO theory, the diffracted rays are introduced with the Geometrical Theory of Diffraction (GTD) and its uniform extension, the Uniform GTD (UTD). The purpose of these rays is to remove the field discontinuities and to introduce proper field corrections, especially in the zero-field regions predicted by GO [28–30]. This simulation technique is optimal in terms of accuracy and required calculation time [31].

**Figure 5.**
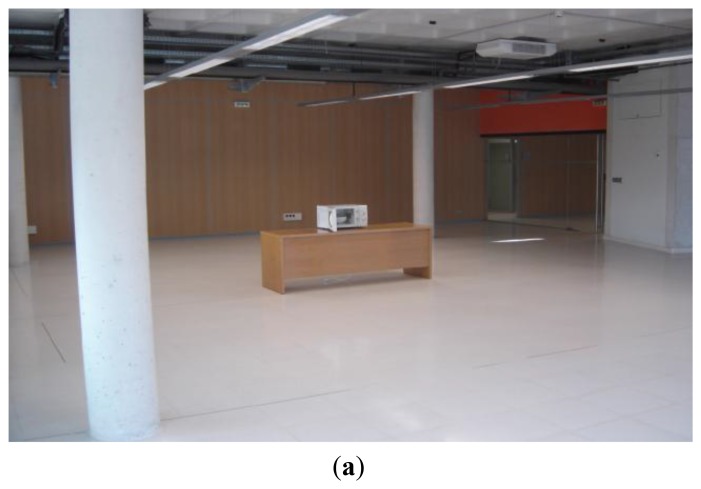

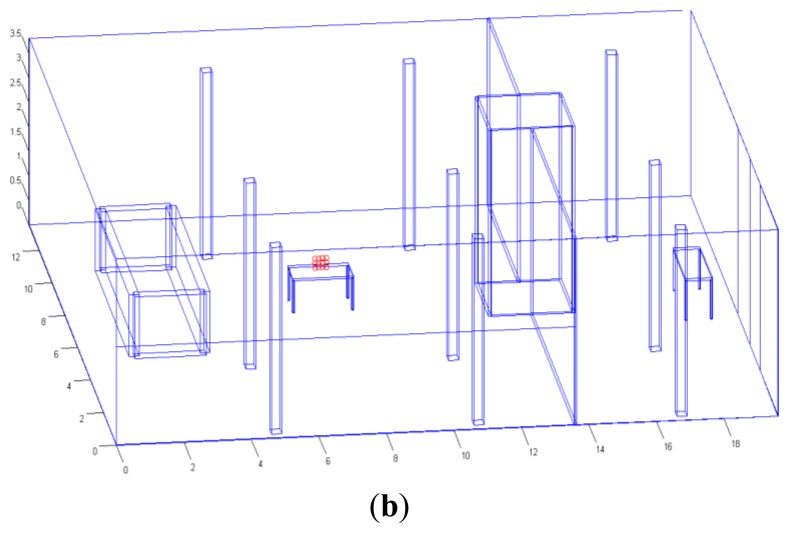
Indoor scenario of ray launching simulation. (**a**) Real scenario. (**b**) Schematic scenario in the 3D ray launching simulation code.

In this work, to model the behavior of the radiated interfering fields caused by power leakage from the microwave oven, equivalent antennas have been calculated using the electric field data obtained outside the microwave oven by the full wave electromagnetic simulation results obtained in the previous section. These equivalent antennas have been introduced in a ray launching algorithm to predict the received power in different points of the considered scenario (Figure 5(a)). The implemented in-house 3D ray launching algorithm is based on Matlab programming environment. Several transmitters can be placed within an indoor scenario, in which power is modeled as a finite number of launched rays within a solid angle. The principle of ray launching method is that a ray is launched from the transmitting antenna and it is traced to see if hits any object or is received by the receiving antenna. When an object is hit, reflection, transmission or diffraction will occur, depending on the geometry and the electric properties of the object. When a ray is received by a receiving antenna, the electric field (power) associated with the ray is calculated. Parameters such as frequency of operation, radiation pattern of the antennas, number of multipath reflections, separation angle between rays and cuboid dimension are introduced, as well as the material properties for all of the elements within the scenario, including the dielectric constant and the loss tangent at the frequency range of operation of the system under analysis. Table 2 shows the parameters used in the ray launching simulation, which have been selected after parametric analysis in order to optimize computational cost and achieve adequate accuracy of the simulation results.

To analyze the impact of the electromagnetic propagation around the microwave oven in the considered scenario (Figure 5), the equivalent transmitter antennas have been introduced in the ray launching simulator. Figure 5(b) shows the schematic of the indoor scenario implemented in the simulator. The red point represents the microwave oven.

**Figure 6.**
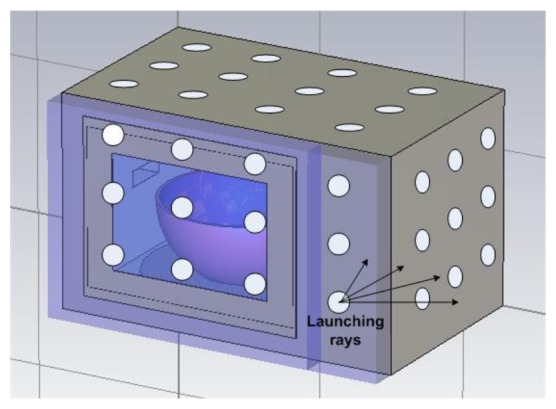
Schematic of the equivalent arrays of transmitter antennas in the microwave oven.

Several equivalent arrays of antennas have been calculated for the front plane (*i.e.*, location of the front door), the backplane, the lateral planes and the upper surface of the microwave oven. These equivalent antennas, which emulate the original simulation source, have been chosen taking into account the surface current distributions obtained in the previous section with full wave simulation. These currents, which are the source of radiated fields, are then mapped into isotropic radiating sources within the half outer plane (*i.e.*, no radiation towards the inner part of the microwave oven, since we are interested in interference due to leakage), in order to simplify the final interference model of the microwave oven. Figure 6 represents the microwave oven with the equivalent arrays of antennas in the different planes.

For the front plane, backplane and upper plane of the microwave oven, arrays of twelve equivalent antennas with different transmitter power each have been introduced to the simulator and the considered array for lateral planes has nine equivalent antennas with different power level (depicted in Figure 6). The transmission power of these antennas has been calculated with the results of the electric field of the different planes taken from the 3D electromagnetic simulation performed in the previous section. This transmitted power has been normalized by 800 W to consider the real transmitter power of the microwave oven. For the receiver, an omni-directional 7dBi indoor antenna has been considered, which provides a 360° horizontal beam width for −3dB and vertical beam width of 23° (which corresponds to an antenna model OAN-1070 from LevelOne).

**Figure 7.**
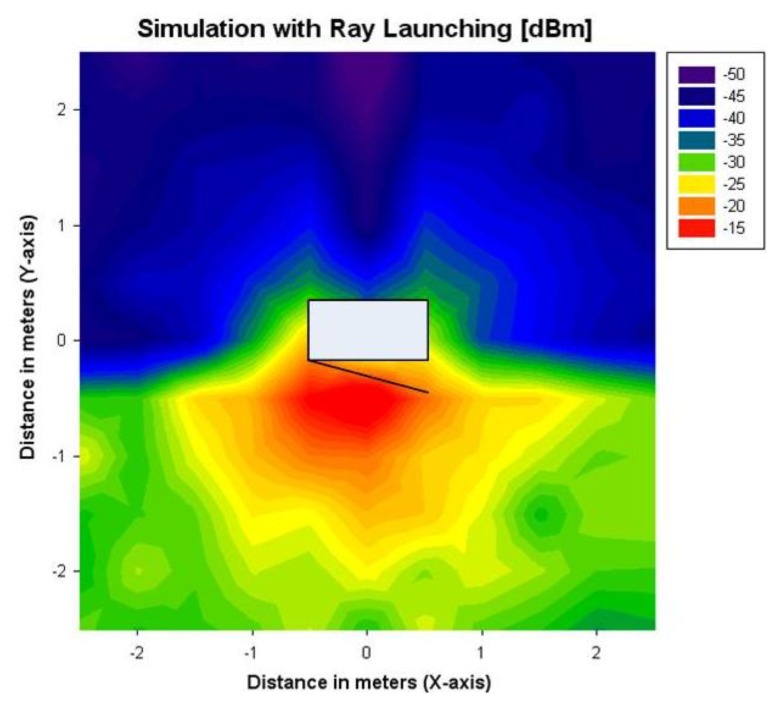
Ray launching simulation results for a height of 0.7 m. The representation of the front door is included in order to indicate the relative position of the microwave oven in the simulation scenario.

Figure 7 shows the obtained ray launching simulation results with the different arrays of equivalent transmitter antennas. As expected, radiated power is higher in the front plane of the microwave oven and it is scattered widely around this plane. However, in the back plane there are not many observable differences. This is due to the fact in the 3D electromagnetic simulation of the internal microwave oven, some sources of losses have not been considered (like microwave racks in the back of the oven, manufacturers joint, *etc.*), since such information was not readily available from the specification set.

**Figure 8.**
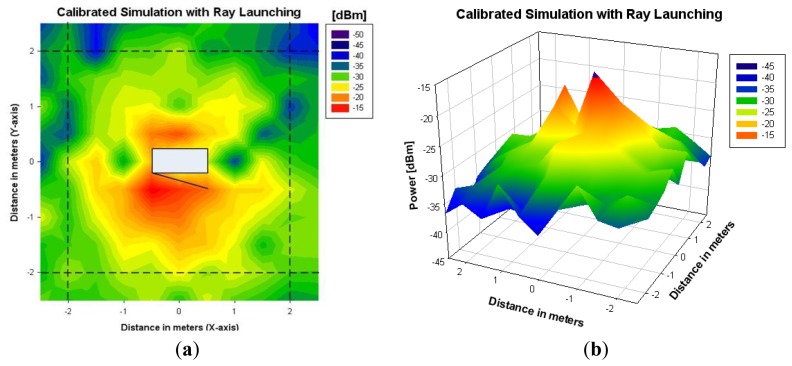
3D Ray launching results for a height of 0.7 m with the losses calibration in the back plane of the microwave. (**a**) Bidimensional plane. (**b**) Tridimensional plane.

**Figure 9.**
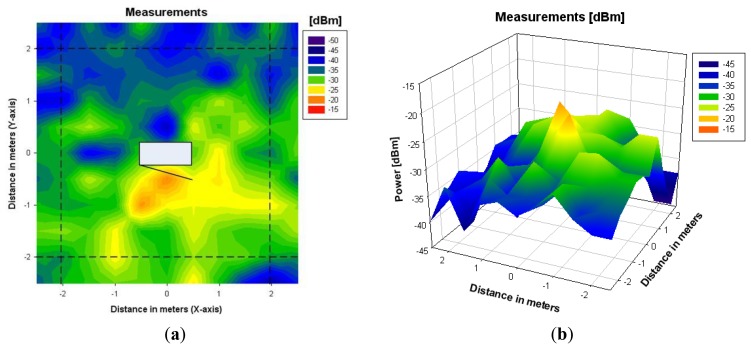
Measurements results for a height of 0.7 m. (**a**) Bidimensional plane. (**b**) Tridimensional volume. The coordinate axis have been placed considering the central point of the microwave oven in the height plane under consideration as the origin of the coordinate system.

To overcome this issue, a calibrated equivalent simulation antenna has been computed from the measurements of the back plane of the scenario. By using the Friis formula and normalizing by the number of launched rays, the calibrated transmitted power has been calculated. Ray launching simulation has been performed again for the back plane with the new calibrated antenna. Figure 8 shows the results obtained after the calibration, whereas Figure 9 shows the values of received measured power in the indoor scenario with the microwave oven under operation, for a coordinate system considering with the origin placed in the central point of the microwave oven.

It can be seen that, after the calibration procedure, measurement and simulation results show good agreement in all of the analyzed planes. Received power decreases with transmitter-receiver distance, having some power peaks in the front plane near the microwave oven. It is also observed that received power is scattered widely in the XY plane. This is due to the fact that the fundamental propagation phenomena in an indoor environment is multipath propagation, which is characterized by the temporal dispersion of the signal and the frequency dispersion due to time dependent variations of the received amplitude [28].

**Figure 10.**
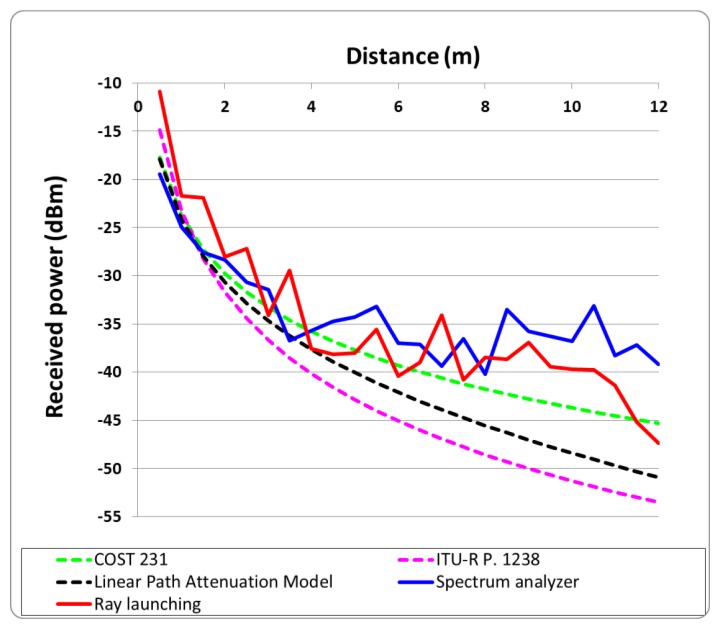
Received power *versus* distance for ray launching simulation, measurement results and estimation by empirical-based propagation models.

To validate the results obtained with the ray launching simulation method, comparison with real measurements and different empirical-based propagation models has been performed. Figure 10 shows the ray launching simulation values and measured power values for different distances from the microwave oven door in a linear distance separating the interference source and potential receiver antennas between 0.5 m and 12 m. These values are accompanied with estimations using different empiric propagation models.

The measurements have been performed with the aid of an Agilent Field Fox N9912A spectrum analyzer. The antenna and the microwave oven have been placed at the same height (0.7 m, which is the level of the table in which the microwave oven was placed within the scenario) and the received power level measurement time for each position was 30 seconds. Due to the fact that the Packet Error Rate (PER), which will be analyzed in the next section, depends directly on the received power, this results can be generalized for all positions within the scenario.

**Figure 11.**
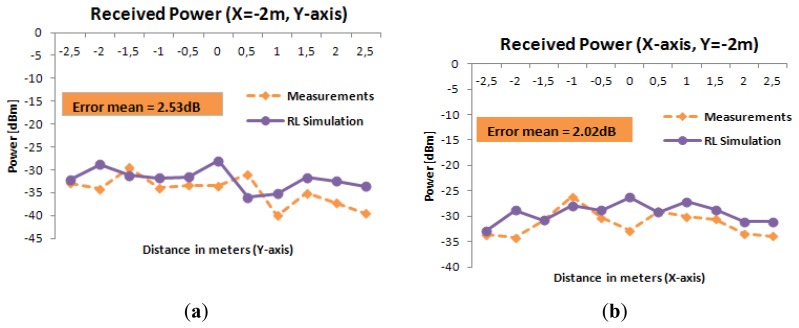

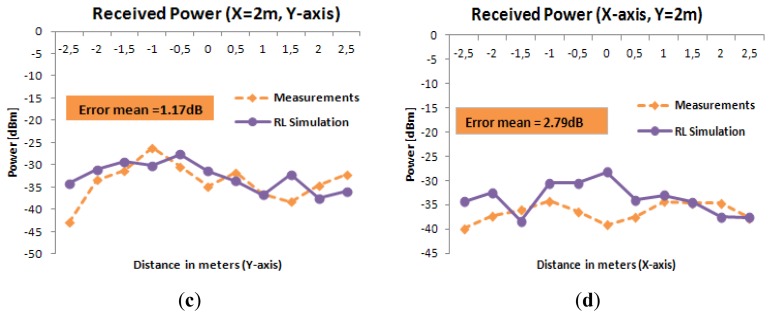
Radial Power for different points of X along the Y-axis ((**a**) and (**c**)) and for different points of Y along the X-axis ((**b**) and (**d**)).

The results shown in Figure 10 show that the ray launching algorithm, which takes into account the effect of multipath propagation, exhibits higher accuracy, with a mean error of 1.29 dB. Linear radials (*i.e.*, linear paths between transmitter and receiver) of received power along different distances around the microwave oven are represented in Figure 11. These radials correspond to the dashed lines depicted in Figures 8(a) and 9(a). From the results it can be seen that simulation and measurements present a mean error around 2dB in all cases, showing good agreement between them.

To summarize, the simulation procedure in this section is the following: once the microwave oven has been simulated, the obtained values of the surface currents are employed to model equivalent radiation sources in the surface of the microwave oven. These sources will now be used as equivalent sources in a 3D ray launching algorithm that we have implemented in-house in our research group in Matlab code. The reason for using this approach is that this type of simulation code can be applied in order to calculate how the radiated leakage fields of the microwave oven can propagate along the entire simulation scenario and not only in the close vicinity of the microwave oven itself. Therefore, we can estimate received interference power levels in the complete room, which is the key information needed in order to consider possible degradation in the operation of the wireless sensor nodes.

## 4. Radio Link Analysis and Results

Once the simulation method has been tested in terms of radiopropagation conditions, radio link interface performance of Wireless Sensor Networks, based on ZigBee XBee transceivers will be analyzed. The scenario where the measurements have been performed is depicted in Figure 5(a). It is located in one of the R&D laboratories at the Public University of Navarre and the model created for the ray launching software can be seen in Figure 5(b). The scenario has the inherent complexity of an indoor location, with interior columns, some furniture elements and walls made of different materials (wood, concrete, bricks, metal and glass). The microwave oven has been located on a wooden table at a height of 0.7, in the location shown in Figure 5(a). The behavior of the microwave oven radiated electromagnetic power leakage has been characterized in first place. Once the nature of the interference has been analyzed, the impact that this interference has on the quality of the ZigBee channels of a set of operating wireless sensors has been measured. The value of PER, *i.e.*, the ratio between packets with errors *vs.* error free packets, has been used as the quality parameter. Due to the spectral width of the microwave oven frequency spectrum, all the ZigBee channels supported by the XBee Pro device have been analyzed (channels 12 to 23 of the 802.15.4 standard).

**Figure 12.**
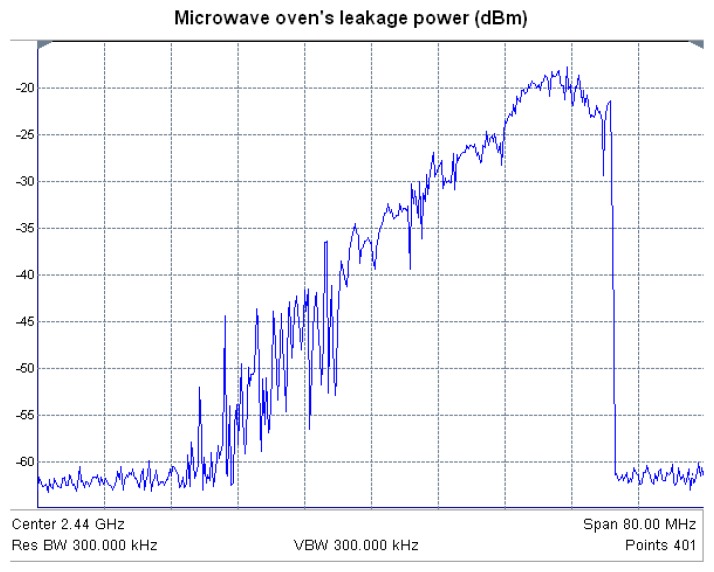
Measurement of the microwave oven leakage power spectrum in 2.36 GHz–2.52 GHz band, obtained from direct spectrum measurement within the indoor scenario.

A portable spectrum analyzer (Agilent Fieldfox N9912A) with a LevelOne OAN-1070 omnidirectional antenna has been used to perform measurements of radiated power. The first measurement performed was the power spectrum when the microwave oven is heating an object. The oven has three operating power levels. Figure 12 shows the measured spectrum of the microwave oven operating at maximum power, in which the interference level will be the highest. The distance from the receiving antenna coupled to the spectrum analyzer to the microwave oven front door is 10 cm and both are placed at the same height. It can be seen that the microwave oven radiates with significant power levels in almost all the 2.4 GHz ISM band, becoming this leakage a significant source of interference to wireless networks [32].

**Figure 13.**
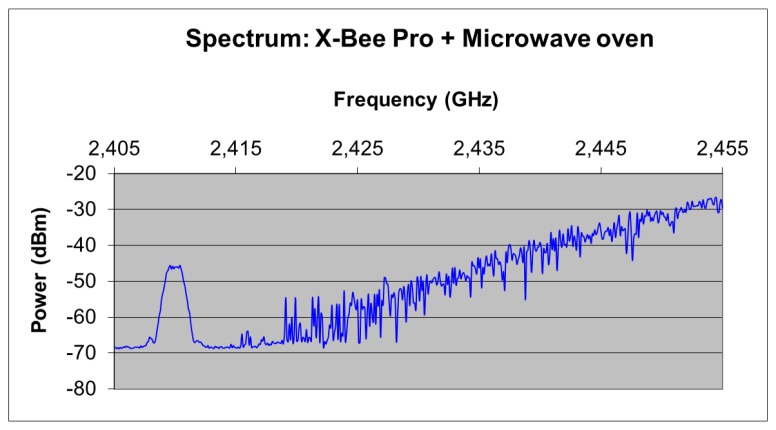
Measurement of ZigBee channel 12 (given by the peak visible on the left hand side) and microwave oven leakage spectrum.

Figure 13 shows the frequency spectrum corresponding to ZigBee channel number 12 alongside the oven leakage power spectrum. As it can be seen, the leakage power level can be higher than the received ZigBee power within the operating range of the sensors. Therefore, the quality of ZigBee channels will be affected when both RF emissions (Wireless Sensor Network and microwave interference spectrum) overlap. This occurs in practically all the ZigBee channels measured in this paper (channels 12 to 23 of the 802.15.4 standard).

**Figure 14.**
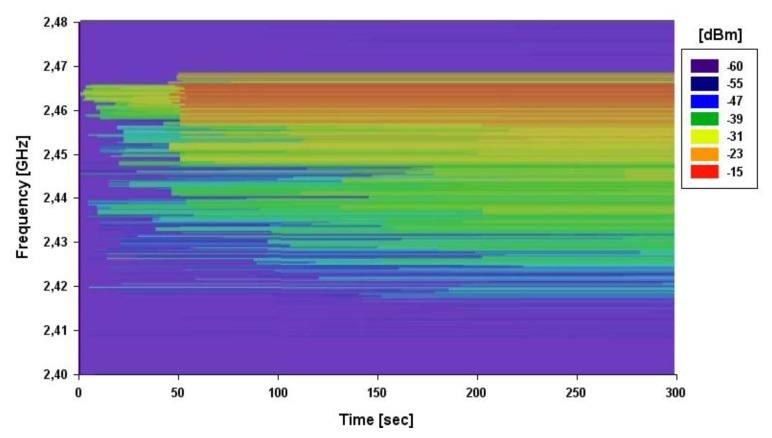
Measured Spectrogram in 2.4 GHz ISM band, corresponding to detected leakage power within the measurement scenario.

Another aspect to consider is the fact that the microwave oven leakage spectrum has a time dependent power distribution. Taking this time dependent nature into account, a spectrogram using the max hold method (*i.e.*, storing the maximum detected power level in the measurement time span) has been obtained (depicted in Figure 14) to gain insight in the power leakage process. The receiver antenna coupled to the portable spectrum analyzer has been placed at the same height of 0.7 m and separated 10 cm from the microwave oven front door. The spectrogram has been obtained by performing measurements in 5 minute intervals once the oven starts heating. The result presented in Figure 14 depicts how the highest frequencies are the first to appear and how the lowest ones need more time in order for them to be observed. Therefore, the interference that each ZigBee channel suffers depends on the microwave oven leakage power level and the time that the microwave oven has been operating.

Once the interference levels have been characterized, the performance of the radiolinks between wireless sensors can be analyzed, in the same scenario previously described. The RF modules employed have been Digi XBee Pro models, which can operate using channels 12 to 23 of the IEEE 802.15.4 standard.

In order to determine the radio link quality, two Java-based applications have been developed in house specifically for this purpose. The first one, the transmitter application, sends the number of packets set by the user. Each one of these packets is identified by a sequence number. The receiver application reads the sequence number of the received packets and determines how many packets have been lost in order to calculate the PER. A receiver ZigBee device has been placed at the same height as the microwave oven and 50 cm away in front of the front door. The transmitter device is placed also at the same height and in front of the microwave oven, but 12 meters away. Because of the irregular radiation diagram of the integrated antennas of XBee Pro modules, care has been taken in maintaining the same antenna orientation in all measurements that have been performed.

**Figure 15.**
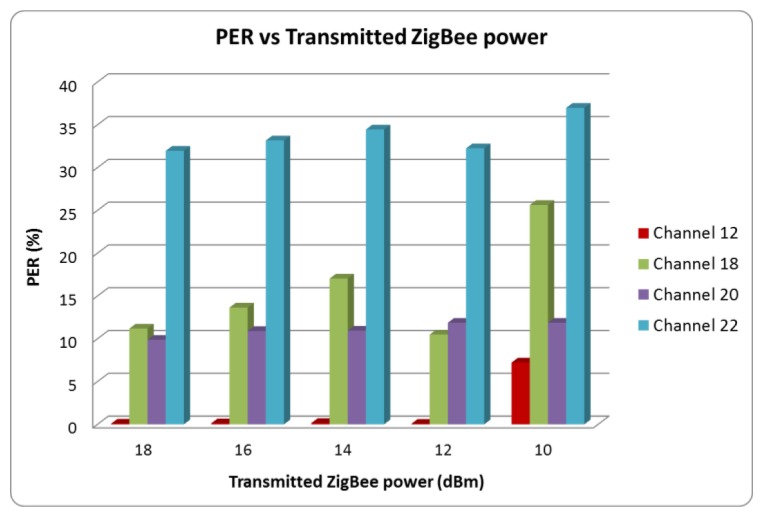
Measured PER values *versus* transmitted power for ZigBee channel 12, 18, 20 and 22.

The PER value has been calculated considering 50,000 transmitted packets for each of the 802.15.4 channels (12 to 23) and each of the available transmission power levels (18, 16, 14, 12 and 10 dBm). Figure 15 shows the measured values of PER for channels 12, 18, 20 and 22, considering different transmitted power levels. As expected, PER increases as the transmitted power level is reduced, due to decrease in Signal to Noise ratio derived from lower available TX power. Channel 12, as can be seen in Figure 13, does not overlap with the microwave oven leakage power spectrum and the measured PER value is very low in this case. For channels located at higher frequencies, an increase of PER can be seen. This is due to higher power level of the microwave oven leakage in those frequencies (depicted in Figure 12). It is worth noting, however that due to the time dependent nature of the interference, there are intermediate cases, *i.e.*, channel 20 where overall interference levels are lower.

**Figure 16.**
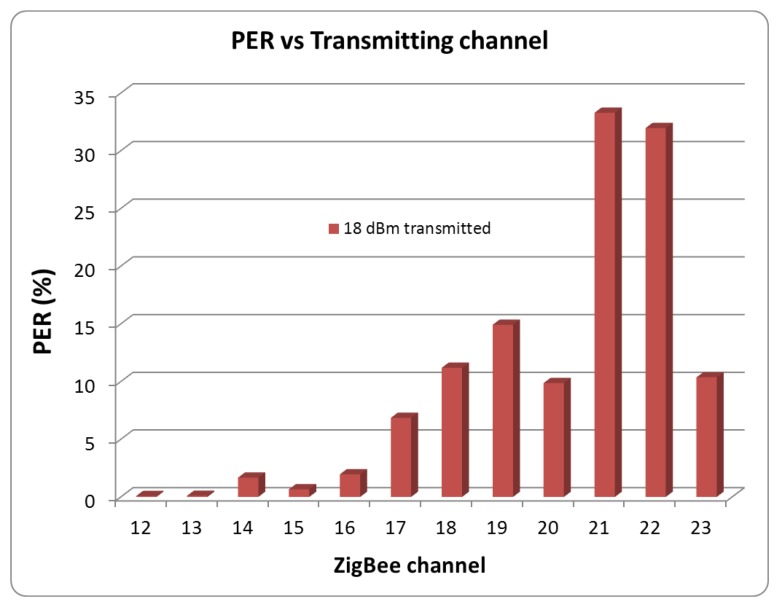
Measured PER for each ZigBee channel, transmitting 18 dBm.

**Figure 17.**
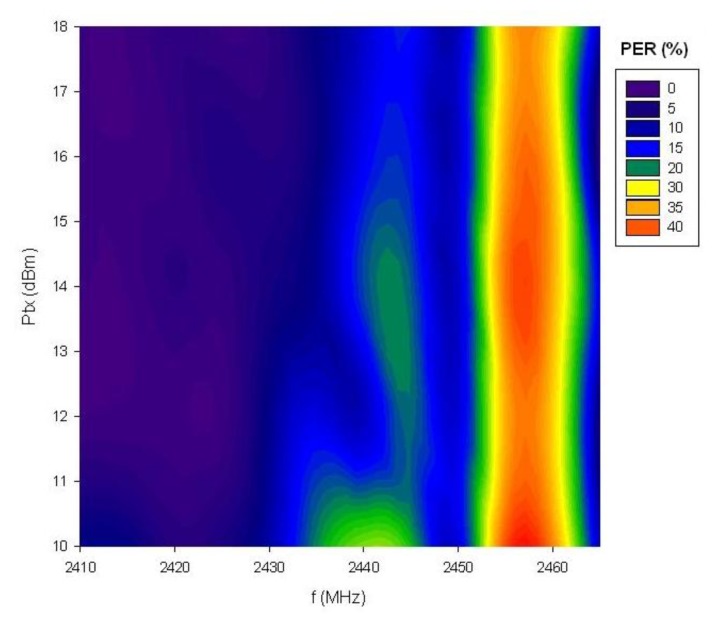
Packet Error Rate measurements (%) as a function of frequency and transmission power.

Figure 16 shows the dependence of PER levels on the selected ZigBee channel when the interference due to microwave oven power leakage appears. The measured values have been taken with the maximum transmission power level of the XBee Pro modules (18 dBm). The same measurements have been taken for all the transmission power levels and the results are channel dependent in all cases, as can be graphically stated in Figure 17. Due to the fact that interference levels due to leakage increase as the channel frequency increases, the Signal to Noise ratio will decrease for the same level of transmit power. Therefore, the direct consequence is an increase of the PER ratio as the channel number is increased, leading to lower radio link quality.

To summarize, the measurement procedure employed has been the following:

- Radio channel measurements in the operating frequency band of the wireless sensors. Measurements were performed with the aid of an Agilent N9912A portable spectrum analyzer, coupled to a small vertical monopole.
- Spectrogram, which is recorded in order to analyze the frequency spectrum variation as a function of time. This is due to the fact that performing previous radiochannel measurements, new frequency components in the spectrum would appear at different time intervals.
- PER measurements of the wireless sensors deployed in a conventional indoor scenario, with a microwave oven under operation. In this case, a Java application has been implemented in-house in our research group in order to obtain the PER measurements. The programmed application employs a Linux rxtxcomm library in order to enable a communication port from the UART port of the sensor to the USB port of the laptop computer in which the application is running. Transmission cycles of data packets were programmed through Attention (AT) commands given by the Java application to the sensor and the received packets were stored in the laptop computer.

## 5. Conclusions

In this work, the impact of a potential source of interference in the deployment of a Wireless Sensor Network and wireless devices operating in the 2.4 GHz band in a complex indoor scenario has been analyzed. This potential source of interference tested is a domestic microwave oven, with ZigBee sensor nodes operating within the influence region of the microwave oven. In order to fully characterize the interference power within the complete volume of the indoor scenario, a combined simulation approach has been adopted in order to reduce computational complexity of the simulation and hence allow analyzing in detail large scenarios. The equivalent distribution of currents on the surface of the microwave oven has been obtained by means of full wave electromagnetic simulation results, which have been combined with an in-house 3D ray launching simulation code by definition of equivalent radiation sources. Simulation as well as measurement results show good agreement, indicating the presence of interference levels within the operation region of the Wireless Sensor Network.

The effect on wireless sensors has been analyzed by performing radiolink measurements on ZigBee based XBee nodes. Due to the time-dependent nature of the interference produced by the microwave oven leakage, the election of the wireless channel has a strong impact on the radiolink channel quality and hence the overall performance of the WSN.

The analysis of potential interference sources taking into account the topology and morphology of complex indoor scenarios can aid in the optimal deployment strategies of Wireless Sensor Networks, coexistent with other wireless elements, in order to minimize energy consumption while enhancing performance in terms of quality as well as overall throughput.

## Figures and Tables

**Table 1.** Materials in the cst microwave schematic.

**Material**	**ε (F/m)**	**μ (H/m)**
Glass (Pyrex) (lossy)	4.82	1
Plexiglas	3.6	1
Porcelain	6	1
Water	81	1

**Table 2.** Parameters in the ray launching simulation.

Frequency	24 GHz
Cuboids resolution	7.5 cm
Vertical plane angle resolution Δ*θ*	1°
Horizontal plane angle resolution Δφ	1°
Reflections	5

## References

[1] Wen Y., Zhang X. Radiation Characteristics of Microwave Ovens.

[2] Wen Y., Zhang L., Liu C., Zhang X. Measurement and Calculation of the Radiation Characteristics of Microwave Ovens.

[3] Matsumoto K., Hashimoto O., Wada K. An Efficient Analysis on Door Structure of a Microwave Oven Using Combined Waves of Higher Order Modes.

[4] Kusama Y., Hashimoto O., Makida M. (2002). Analysis of door seal structure of microwave oven with consideration of higher modes by the FDTD method. Electron. Comm. Jpn..

[5] Soltysiak M., Celuch M., Erle U. Measured and Simulated Frequency Spectra of the Household Microwave Oven.

[6] Matsumoto Y., Takeuchi M., Fujii k., Sugiura A., Yamanaka Y. (2005). Performance analysis of interference problems involving DS-SS WLAN systems and microwave ovens. IEEE Trans. Electromagn. Compat..

[7] Simek M., Fuchs M., Mraz L., Moravek P., Botta M. Measurement of lowPAN Network Coexistence with Home Microwave Appliances in Laboratory and Home Environments.

[8] Taher T.M., Misurac M.J., LoCicero J.L., Ucci D.R. Microwave Oven Signal Interference and Mitigation for Wi-Fi Communication Systems.

[9] Rondeau T.W., D'Souza M.F., Sweeney D.G. (2004). Residential microwave oven interference on Bluetooth data performance. IEEE Trans. Consum. Electron..

[10] Mingxin N., Ling L. Simulation of Microwave Oven Interference on Digital Radio Communication Systems.

[11] Zualkernan A., Al-Ali A.R., Jabbar M.A., Zabalawi I., Wasfy A. (2009). InfoPods: Zigbee-Based remote information monitoring devices for Smart-Homes. IEEE Trans. Consum. Electron..

[12] Bellido-Outeirino F.J., Flores-Arias J.M., Domingo-Perez F., Gil-de-Castro A., Moreno-Munoz A. (2012). Building lighting automation through the integration of DALI with wireless sensor networks. IEEE Trans. Consum. Electron..

[13] Yan H., Huo H., Xu Y., Gidlund M. (2010). Wireless Sensor Network Based E-Health System—Implementation and Experimental Results. IEEE Trans. Consum. Electron..

[14] Gill K., Yang S., Yao F., Lu X. (2009). A ZigBee-based home automation system. IEEE Trans. Consum. Electron..

[15] Han J., Choi C., Lee I. (2011). More efficient home energy management system based on ZigBee communication and infrared remote controls. IEEE Trans. Consum. Electron..

[16] Han D.-M., Lim J.-H. (2010). Smart home energy management system using IEEE 802.15.4 and ZigBee. IEEE Trans. Consum. Electron..

[17] Han D.-M., Lim J.-H. (2010). Design and implementation of smart home energy management systems based on ZigBee. IEEE Trans. Consum. Electron..

[18] Huo H., Xu Y., Bilen C.C., Zhang H. Coexistence Issues of 2.4GHz Sensor Networks with other RF Devices at Home.

[19] Guo W., Healy W.M., Zhou M. An Experimental Study of Interference Impacts on ZigBee-Based Wireless Communication inside Buildings.

[20] Guo W., Healy W.M., Zhou M. Performance Measurement and Analysis of Low Data Rate Wireless Communication under Interference Sources in Buildings.

[21] Guo W., Healy W.M., Zhou M. (2012). Impacts of 2.4-GHz ISM band interference on IEEE 802.15.4 wireless sensor network reliability in buildings. IEEE Tran. Instrum. Meas..

[22] Iwabuchi K., Kubota T., Kashiwa T. (1996). Analysis of Electromagnetic Fields in a Mass-Produced Microwave Oven Using the Finite-Difference Time-Domain Method. J. Microwave Power Electromagn..

[23] Han J., Qing Z. (2009). Simulation and experimental method for microwave oven. J. Electron. Sci. Technol. China.

[24] Monteiro J., Costa L.C., Valente M.A., Santos T., Sousa J. Simulating the electromagnetic field in microwave ovens.

[25] Sung Y., Lie L., Chian K.S., Fei S., Shan G. A Study of Microwave Curing Process for underfill Used in Flip Chip Packaging. Part 2: 3D FEM Simulation of Microwave Power Distribution Inside Variable Frequency Microwave Oven.

[26] Kiley E.M., Yakovlev V.V. Modeling of Microwave Ovens with Perforated Metal Walls.

[27] Qi J., Kettunen H., Wallen H., Sihvola A. Different Retrieval Methods Based on S-Parameters for the Permittivity of Composites.

[28] Hashemi H. (1993). The indoor radio propagation channel. Proc. IEEE.

[29] Luebbers R.J. (1989). A heuristic UTD slope diffraction coefficient for rough lossy wedges. IEEE Trans. Antennas Propagat..

[30] Luebbers R.J. (1988). Comparison of lossy wedge diffraction coefficients with application to mixed path propagation loss prediction. IEEE Trans. Ant. Prop..

[31] Iskander M.F., Yun Z. (2002). Propagation prediction models for wireless communication systems. IEEE Trans. Microwave Theory.

[32] Zhao Y., Agee B., Reed J.H. Simulation and Measurement of Microwave Oven Leakage for 802.11 WLAN Interference Management.

